# Elevated expression of mature miR-21 and miR-155 in cancerous gastric tissues from Chinese patients with gastric cancer

**DOI:** 10.1016/S1674-8301(10)60028-0

**Published:** 2010-05

**Authors:** Li Liu, Qi Chen, Rensheng Lai, Xiaobin Wu, Xiaoyu Wu, Fukun Liu, Guohua Xu, Yong Ji

**Affiliations:** a Atherosclerosis Research Center, Nanjing Medical University, Nanjing 210029, Jiangsu Province, China; b Laboratory of Cytology & Molecules/Department of Pathology, the Affiliated Hospital of Nanjing University of Traditional Chinese Medicine, Nanjing 210029, Jiangsu Province, China; c Department of Surgery, the Affiliated Hospital of Nanjing University of Traditional Chinese Medicine, Nanjing 210029, Jiangsu Province, China; d Department of Biology, Jiangsu Institute of Education, Nanjing 210013, Jiangsu Province, China

**Keywords:** MicroRNA, expression, gastric cancer

## Abstract

**Objective:**

MicroRNA (miRNA) expression is deregulated in many types of human cancers. We sought to investigate the expression patterns of the miRNAs, miR-21, miR-145 and miR-155 in sporadic gastric cancer in a Chinese population.

**Methods:**

Total RNA was extracted from archived gastric cancer tissues and adjacent non-cancerous tissues from 20 pairs of paraffin-embedded specimens. Expression levels of miR-21, miR-145 and miR-155 were detected by quantitative reverse transcriptase PCR using a specific stem-loop primer, with U6 as the internal reference gene.

**Results:**

The expression of miR-21 and miR-155 in gastric cancer samples was significantly higher than in paired non-cancerous samples (*P* < 0.05). There was no statistically significant difference in expression levels of miR-145 between cancerous and non-cancerous tissues (*P* > 0.05).

**Conclusion:**

In Chinese sporadic gastric cancer tissues, the expressions of the oncogenic miR-21 and miR-155 were significantly up-regulated, while the expression of the tumor suppressor miR-145 was decreased, although this decrease was not statistically significant. Thus there is specificity in the miRNA expression pattern in gastric cancers in the Chinese population.

## INTRODUCTION

MicroRNAs (miRNAs) are small, non-coding RNAs of 19-24 nucleotides in length that were first reported by Lee and colleagues[1]. Sequences of miRNAs are highly conserved[2], and they regulate gene expression at a post-transcriptional level, most commonly by inhibiting mRNA translation or promoting mRNA degradation[3]. miRNAs are frequently misregulated in human malignancies[4]. Over the past 10 years, many findings have strongly supported a role for miRNAs in the regulation of crucial processes such as cell proliferation[5], apoptosis[6], development[7], differentiation[8] and metabolism[9]. Therefore, miRNAs dysfunction may contribute to a variety of human diseases such as cancer, cardiovascular disease, liver disease, immune dysfunction and metabolic disorders[10]. The first evidence that miRNAs were involved in cancer came from the finding that miR-15a and miR-16-1 were down-regulated or deleted in most patients with chronic lymphocytic leukemia[11]. In tumor diseases, miRNAs may act as oncogenes or tumor suppressor genes, and sometimes both[12],[13].

miRNAs may be used as molecular markers for early diagnosis and prognosis of cancer. In a study by Yanihara *et al*[14], microarray analysis identified unique miRNA expression profiles which could discriminate between cancerous and non-cancerous lung tissue. Similarly, in breast tumors a reduced expression of let-7a correlated with bad prognosis, characterized by lymph node metastasis and a high proliferative index[15]. These findings demonstrate that expression analyses of miRNAs in neoplastic cells are of significance for diagnosis of tumor malignancy and subsequent patient outcome.

According to data from 2002, stomach cancer was the second most common form of cancer worldwide and the second most common cause of cancerous mortality. Almost two-thirds of cases occurred in developing countries, of which 42% occurred in China alone[16]. These data highlight the current need for further research into gastric cancer, particularly in China. While some studies on the relationship between miRNAs and gastric cancer have recently been done (reviewed by Saito *et al*[17]), little work[18]-[21] has been carried out in China. It is important to note that differential gene expression may occur among different ethnic groups[22],[23] as well as within ethnic groups of different spatial localization[24] and within different individuals of the same race[25]. From a Chinese health perspective, studies into the relationship between miRNAs and Chinese sporadic gastric cancer could provide valuable information.

In this paper, two potential oncogenic miRNAs (miR-21 and miR-155) and one potential tumor suppressor miRNA (miR-145)[12],[17] were chosen to investigate the relationship between miRNA expression level and Chinese gastric cancer, with the aim of elucidating possible differences in miRNA expression patterns within the Chinese population.

## MATERIALS AND METHODS

### Samples

All samples were obtained from patients who had undergone surgical gastric resection at the Affiliated Hospital of Nanjing University of Traditional Chinese Medicine. Twenty paired samples were selected randomly and examined independently by two experienced pathologists. The samples were gastric cancer tissues and adjacent non-cancerous tissues in the same patient. Patients all came from Jiangsu Province and Anhui Province in China and each patient gave his/her informed consent. A summary of the pathological characteristics of these patients is given in ***Table 1***. The average age of the patients was 51.9 years (range 40-71).

**Table 1:** Summary of pathological characteristics of samples

Sample	Pathological chracteristics	No.(%)
Age (years old)	<50	8(40)
	>50	12(60)
Laren classification	Intestinal	11 (55)
	Diffuse	9 (45)
WHO classification	Tubular adenocarcinoma	15 (75)
	Signet-ring cell carcinoma	4 (20)
	Mucinous adenocarcinoma	1 (5)
Differentiation	Mod-well+ Mod	4 (20)
	Mod-poor	4 (20)
	Poor	12 (60)
TNM Stage	I	3 (15)
	II	3 (15)
	III	12 (60)
	IV	2 (10)
Lymph node status	N0	6 (30)
	N1	7 (35)
	N2+N3	7 (35)
*H. pylori infection*	-	9 (45)
	+	10 (50)

### Total RNA extraction

RNA was isolated from formaldehyde-fixed paraffin-embedded (FFPE) tissues with RecoverAll™ Total Nucleic Acid Isolation Kit (Ambion USA) which is designed to extract total nucleic acids (RNA, miRNA, and DNA) from FFPE tissues. Up to four 20 µm sections, or up to 35 mg of unsectioned core samples were processed per reaction. Paraffin was removed with 100% xylene, and the pellet was washed twice with 1 ml of 100% ethanol and air-dried. After addition of 400 µl digestion buffer and 4 µl protease, the sample was incubated at 50 °C for 3 h. Isolation additive (480 µl) and ethanol (1.1 ml) were added, and the samples passed through a filter cartridge. The filter cartridge was washed with 700 µl wash 1 solution and 500 µl wash 2/3 solution, and then was centrifuged to remove residual fluid. DNase mix was added to each filter cartridge and incubated for 30 min. After being washed once with 700 µl wash 1 solution and twice with 500 µl of wash 2/3 solution, the sample was centrifuged to remove residual fluid. Samples were eluted in 60 µl elution solution at room temperature. RNA concentration and purity were quantified by spectrophotometry (A260/280 value of about 2.0).

### Reverse transcription and real-time PCR

Quantification of expression levels of miRNAs was done using a two-step RT-PCR method. The PCR primer pairs for miR-21, miR-145 and miR-155 were obtained commercially from Applied Biosystems (Applied Biosystems, ABI, USA). cDNA was reverse transcribed from total RNA samples using specific miRNA primers from the TaqMan MicroRNA Assays (ABI, USA) and reagents from the TaqMan® MicroRNA Reverse Transcription Kit (ABI, USA). Reverse transcriptase reactions contained 20 ng RNA samples, 1.5 µl 10×RT buffer, 0.15 µl dNTP mix (100 mM), 0.19 µl RNase inhibitor (20 U/µl), 1 µl Multiscribe^TM^ RT enzyme (50 U/µl), and 3 µl stem and loop RT primer. The 15 µl reactions were incubated for 30 min at 16 °C, 30 min at 42 °C, 5 min at 8 5 °C, and held at 4 °C.

Real-time PCR was performed using the TaqMan MicroRNA Assay together with the TaqMan® Universal PCR Master Mix (ABI, USA). Twenty mocroliter real-time PCR reactions included 1.33 µl RT product dilution (1:15), 10 µl 2×Taqman® Universial PCR Master Mix, 1 µl 20×PCR primer (TaqMan® MicroRNA Assays; ABI), and 7.67 µl nuclease-free water. U6 was used as an internal control to normalize the expression levels of target genes by correcting differences in the amount of cDNA loaded into PCR reactions. The real-time PCR began with an initial denaturation step at 95 °C for 10 min, 50 cycles of 15 s at 95 °C, 1 min at 60 °C. All reactions were run in triplicate using the ABI 7900 HT Fast Real-Time PCR System with SDS software (version 2.3, ABI), and average threshold cycle number (Ct) data of each miRNA was analyzed with RQ Manager software (Version 1.2, ABI).

The ΔCt method[26] and 2^−ΔΔCt^ method[27] were used for analysis. The ΔCt value was the difference between the Ct value of the specific miRNA and the Ct value of U6, ΔCt = Ct (miRNA)–Ct (U6). ΔΔC t= ΔCt (cancer tissues) –ΔCt (non-tumor tissues). The value of 2^−ΔCt^ represents miRNA expression or content of each sample[28]. The value of 2^−ΔΔCt^ represents the expression relative quotient (RQ) of target gene to the control gene. Here, RQ showed the value of miRNA expression ratio in cancerous tissue to that in adjacent noncancerous tissue. An RQ <1 indicates that expression levels of miRNAs in cancerous tissue are lower than that in non-cancerous tissue. Conversely, an RQ >1 indicates higher miRNA expression in cancerous tissue compared to non-cancerous tissue.

### Statistical analysis

The data were analyzed with SPSS software (Version 12.0, SPSS Inc., USA). The paired samples *t* test was used to compare the content of miRNAs in cancerous tissues and adjacent normal tissues. The Pearson test and Spearman rank test were applied to analyze the correlation of RQ values for the three miRNAs. The Mann-Whitney U test and Kruskal-Wallis test were used to evaluate the correlation between miRNA change and clinicopathological parameters. *P* < 0.05 was considered to be statistically significant.

## RESULTS

### Ct values of three miRNAs from the paired gastric tissue samples

**Fig. 1:**
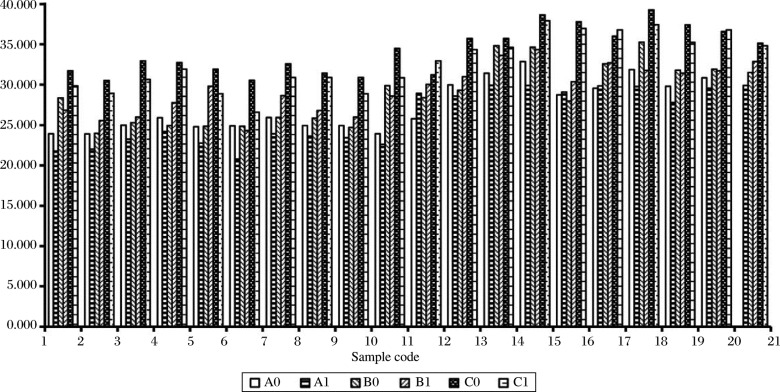
Ct values of miR-21, -145 and -155 in human gastric tumor tissue (TT) and adjacent non-tumor tissue (NTT) by qRT-PCR. Expression of miR-21, -145 and -155 in human gastric cancer FFPE tissues were examined by qRT-PCR using real-time PCR. Twenty cases of gastric cancers were examined. The paired samples from tumor tissue and the adjacent non-tumor tissue in the same patient were examined. A0: miR-21 in NTT; A1: miR-21 in TT; B0: miR-145 in NTT; B1: miR-145 in TT; C0: miR-153 in NTT; C1: miR-153 in TT.

***Fig. 1*** shows the Ct values of miRNAs obtained from cancerous and adjacent non-cancerous gastric tissues from the sample population. For miR-21, the Ct values for cancerous and non-cancerous tissues ranged from 20.808 to 29.920, and from 23.905 to 32.859 respectively. The Ct values for miR-145 ranged from 24.343 to 34.352 in cancerous tissues and from 24.005 to 35.291 in non-cancerous tissues. The Ct values for miR-155 ranged from 26.674 to 37.953 in cancerous tissues and from 30.533 to 39.270 in normal tissues. In each cancerous tissue sample of the 20 cases, the Ct value of miR-21 was less than that of miR-155. In non-cancerous tissues of the 20 cases, the Ct value of miR-145 was less than that of miR-155. The Ct value of miR-21 was less than that of miR-155, except one case (data test undetermined) in non-cancerous tissues, and the Ct value of miR-145 was less than that of miR-155 in the cancerous tissue of 19 cases.

### miR-21 expression of paired cancerous and non-cancerous samples

**Fig. 2:**
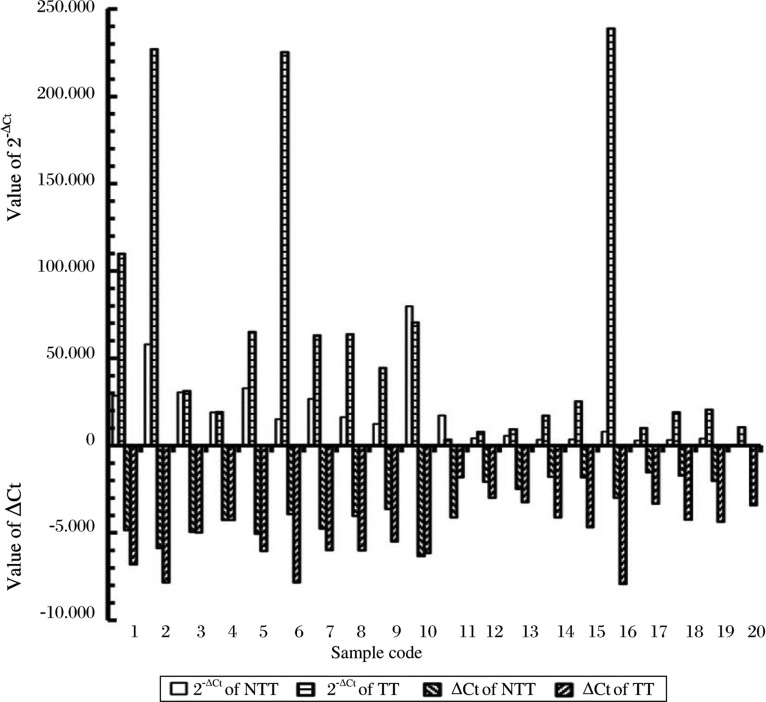
Value of ΔCt and the respective 2^−ΔCt^ of miR-21 in gastric tumor tissue (TT) and non-tumor tissue (NTT). The ΔCt range was from -6.318 to -1.707 and the 2^−ΔCt^ range was from 3.265 to 79.804 in non-tumor tissues, while the ΔCt range was from -7.900 to -1.814 and 2^−ΔCt^ range was from 3.517 to 238.870 in cancerous tissues.

-ΔCt and 2^−ΔCt^ values of miR-21 of non-cancerous and cancerous tissue are presented in ***Fig. 2***. ΔCt = Ct value of miR-21–Ct value of U6. ***Fig. 2*** shows that the miR-21 contents of 17 of 19 pairs (89.5%) were elevated in cancerous tissues compared to adjacent non-cancerous gastric tissue. The average ΔCt and the average miR-21 content (2^−ΔCt^) for non-cancerous and cancerous gastric tissues were -3.580 and -5.149, as well as 19.555 and 66.540, respectively (***Table 2***). The content of expressed miR-21 in cancerous tissues was 3.403 fold higher than in non-cancerous tissues (Fold = 2^−AaverageΔCt (cancer tissues)^ / 2^−averageΔCt (non-tumor tissues)^, and the content of miR-21 in cancerous tissues was significantly higher than that in non-cancerous tissues (*P* = 0.012) (***Table 2***).

**Table 2:** Content of three miRNAs in non-tumor and cancerous gastric tissues

		Non-tumor	Cancer	P value
miR-21	Average ΔCt	-3.580 ± 1.510	-5.149 ± 1.732	0.012 *
(*n* = 19)	Content =2^−Average ΔCt^	19.555 ± 20.420	66.540 ± 76.957	0.012 *
				
miR-145	Average ΔCt	-2.137 ± 2.308	-1.640 ± 1.708	0.134 *
(*n* = 20)	Content =2^−Average ΔCt^	11.924 ± 15.187	6.215 ± 8.284	0.134 *
				
miR-155	Average ΔCt	3.182 ± 1.639	1.674 ± 1.722	0.022 *
(*n* = 20)	Content =2^−Average ΔCt^	0.171 ± 0.142	0.643 ± 0.906	0.022 *

*Paired Samples t test (2-tailed); ΔCt = Ct (miRNA) – Ct (U6).

### miR-145 expression of paired cancerous and non-cancerous samples

**Fig. 3:**
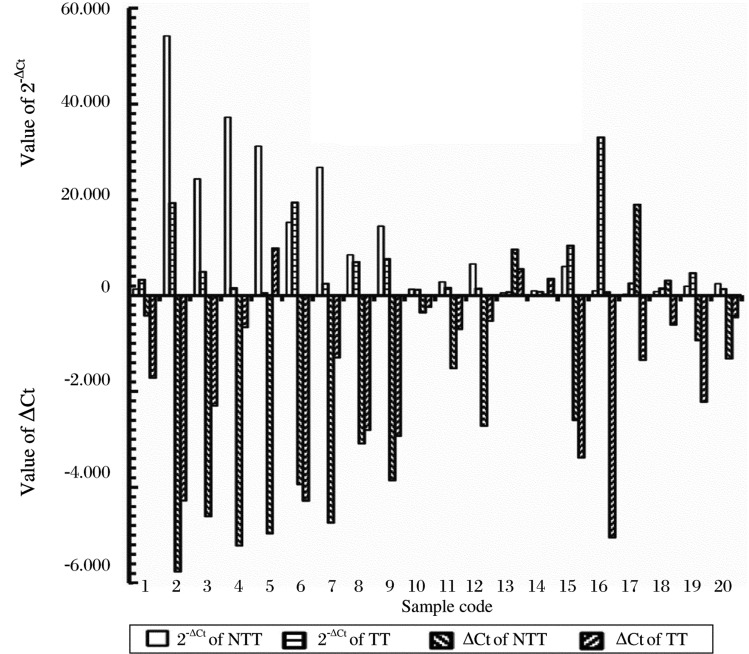
Value of ΔCt and the respective 2^−ΔCt^ of miR-145 in gastric tumor tissue (TT) and non-tumor tissue (NTT). The ΔCt range was from -5.761 to 1.897 and the 2^−ΔCt^ range was from 0.269 to 54.213 in non-tumor tissues. The ΔCt range was from -5.045 to 0.987 and the 2^−ΔCt^ range was from 0.504 to 33.023 in cancerous tissues.

-ΔCt and 2^−ΔCt^ values of miR-145 of non-cancerous and cancerous tissue are presented in ***Fig. 3***. ΔCt = Ct_(miR−145)_–Ct_(U6)_. ***Fig. 3*** shows that the miR-145 content of 12 of 20 pairs (60%) was decreased in cancerous tissues compared to non-cancerous tissues from the same patients. The average ΔCt and the average miR-145 content (2^−ΔCt^) for non-cancerous and cancerous gastric tissues were -2.137 and -1.640, and 11.924 and 6.215, respectively. The expressed miR-145 content in cancerous tissues was 0.521 that in non-cancerous tissues (Fold=2^−AverageΔCt (cancer tissues)^ / 2^−AverageΔCt (non-tumor tissues)^. The content of miR-145 in cancerous tissues was, however, not significantly lower than that in non-cancerous tissues (*P* = 0.134) (***Table 2***). These results mean that miR-145 expressions in non-cancerous and cancerous gastric tissue were not significantly different. But, in general, the expression of miR-145 in tumor tissue (6.215) was lower than that in non-cancerous tissue (11.924).

### miR-155 expression of paired cancerous and non-cancerous samples

**Fig. 4:**
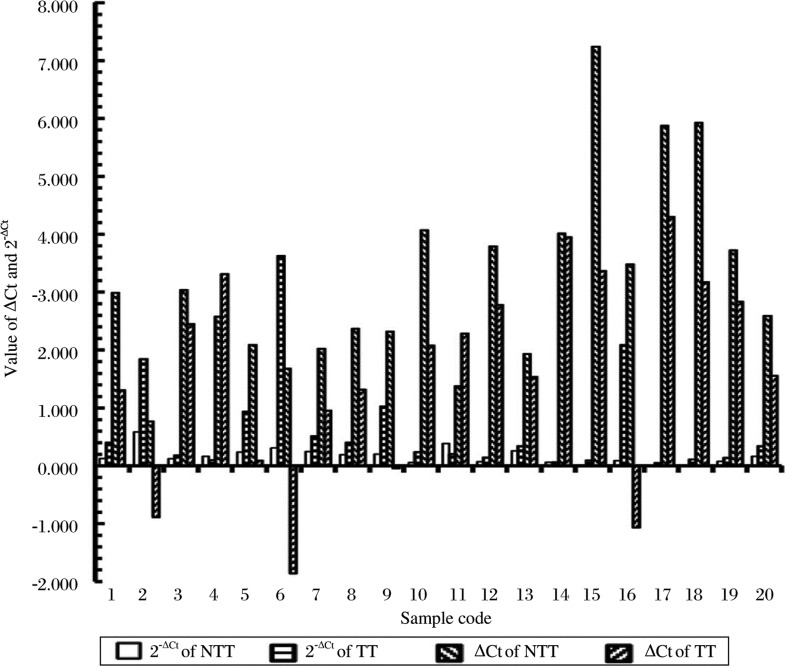
Value of ΔCt and the respective 2^−ΔCt^ of miR-155 in gastric tumor tissue (TT) and non-tumor tissue (NTT). The ΔCt range was from 0.768 to 7.241 and the 2^−ΔCt^ range was from 0.007 to 0.587 in non-tumor tissues. The ΔCt range was from -1.857 to 4.300 and the 2^−ΔCt^ range was from 0.051 to 3.624 in cancerous tissues.

The -ΔCt and 2^−ΔCt^ values of miR-155 of non-cancerous and cancerous tissue are presented in ***Fig. 4***. The ΔCt = Ct_(miR−155)_–Ct_(U6)_ values show that the miR-155 content of 18 of 20 pairs (90%) was up-regulated in cancerous tissues compared to non-cancerous tissues. The average ΔCt and the average miR-155 content (2^−ΔCt^) for non-cancerous and cancerous gastric tissues were 3.182 and 1.674, as well as 0.171 and 0.643, respectively. The expressed miR-155 content in cancerous tissues was 3.760 fold higher than in noncancerous tissues (Fold=2^−AverageΔCt (cancer tissues)^ / 2^−AverageΔCt (non-tumor tissues)^. The content of miR-155 in cancerous tissues was significantly higher than that in non-cancerous tissues (*P* = 0.022) (***Table 2***).

### Correlations among the expression of miR-21, -145, -155

2^−ΔCt^ values of miR-21,-145 and -155 were compared with each other by paired samples *t* test (two-tailed) in cancerous tissues or non-cancerous tissues respectively. In cancerous tissues, the content of miR-21 was significantly higher than that of miR-145 and miR-155 (*P* = 0.001, *P* = 0.001, respectively), and the content of miR-145 was significantly higher than that of miR-155 (*P* = 0.004). In gastric non-cancerous tissues, the content of miR-21 and miR-145 were significantly higher than that of miR-155 (*P* = 0.001, *P*= 0.003, respectively), but there was no difference between the content of miR-21and that of miR-145 (*P* = 0.123).

**Fig. 5:**
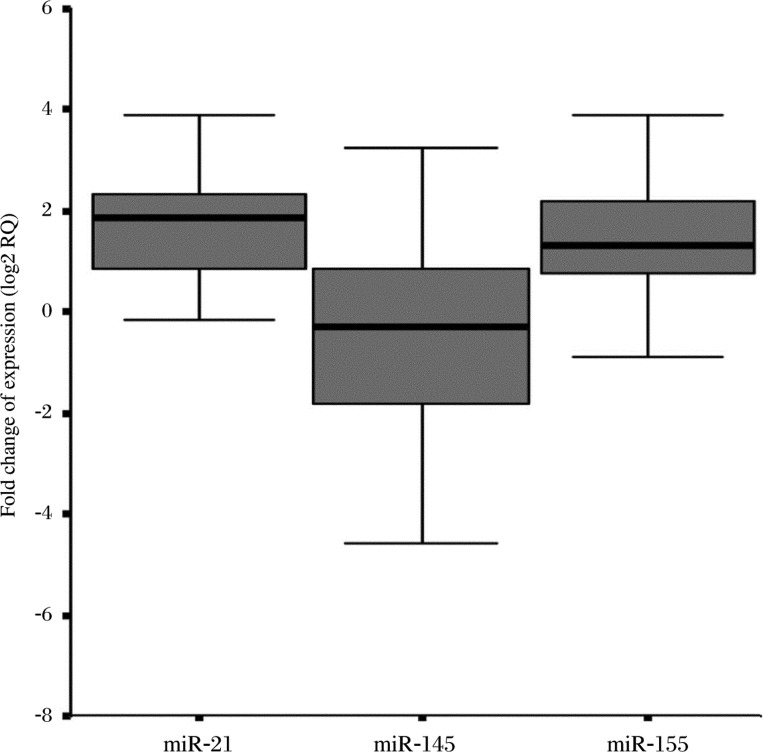
Fold changes of the expression of the three miRNAs. The mean RQ of miR-21 was 5.161±6.908; The mean RQ of miR-145 was 3.014±7.719; The mean RQ of miR-155 was 4.696±5.690. The correlation between RQ of miR-21 and RQ of miR-145: *P* = 0.013, *r* = 0.558 (Spearman Rank test, 2-tailed); The correlation between RQ of miR-21 and RQ of miR-155: *P* = 0.000, *r* = 0.904 (Pearson test, 2-tailed); The correlation between RQ of miR-145 and RQ of miR-155: *P* = 0.110, *r* = 0.368 (Spearman Rank test, 2-tailed).

Fold change (RQ=2^−ΔΔCt^) values of miR-21, -145, -155 are shown in ***Fig. 5***. Spearman rank test data showed that there were significant correlations among the fold changes of miR-21 and miR-145, and miR-21 and miR-155. The results suggest that their expression in gastric cancer may show interactions and the pathways for the regulation of their expression may have affected one another. However, there was no significant correlation between the expression of miR-145 and miR-155.

### Relationships between the expressions of the three miRNAs and the clinicopathological features of the gastric cancers

Associations between the content of miR-21, -145, -155 in gastric tissues and clinicopathological parameters were analyzed by the Mann-Whitney *U* test and the Kruskal-Wallis test. Increased contents of miR-21 and -155, and changes in miR-145 content in gastric cancer were not associated with age (>50 *vs* <50), Lauren classification (intestinal *vs* diffuse), WHO classification (tubular adenocarcinoma, signet-ring cell carcinoma, mucinous adenocarcinoma), differentiation (moderate-well + moderate, moderate-poor, poor), lymph node status (N0, N1, N2+N3), TNM stage (I, II, III, IV), or helicobacter pylori (Hp) infection (negative *vs* positive) (data not shown).

Patients with decreased miR-21 expression in non-cancerous gastric tissue, 1 case of intestinal and 1 case of a diffuse type tumor, patients > 50, or with poorly differentiated tubular adenocarcinomas, or deeply invaded tumors, were associated with lymph node metastasis and Hp infection. All 12 cases of decreased miR-145 expression were found in poorly differentiated tumors, including 6 cases of intestinal-type gastric cancer, 6 cases of diffuse-type gastric cancer, 6 cases of less than 50 years old, 8 cases of tubular adenocarcinoma, 8 cases of lymph node metastasis, 8 cases of Hp infection, and 11 cases of progressive gastric cancer. The exceptional 2 cases of decreased miR-155 expression in cancerous tissue, 1 with lymph node metastasis and the other without, were both from patients more than 50 years old, with an intestinal type tubular adenocarcinoma and were associated with Hp infection.

There was one case in which miR-21, -145 and -155 expressions were all down-regulated in the cancerous tissue, which was a poorly differentiated, TNM stage III intestinal tubular adenocarcinoma, with N2 lymph node metastasis from a patient who was positive for Hp infection. In one case of a mucinous adenocarcinoma the miR-21, -145 and -155 expression levels were much higher than in any other samples and the RQ values were 30.278, 34.636 and 23.382, respectively.

## DISCUSSION

The relationship between alteration in the expression of miRNAs and gastric cancer is a challenging issue for researchers. Zhang *et al*[29] found that the expression level of let-7a miRNA in gastric tumor tissues was significantly reduced when compared to normal tissues in 14 samples from 32 patients. miRNA expression profiling revealed a limited set of miRNAs with altered expression in the multidrug-resistant (MDR) gastric cancer cell line SGC7901/VCR compared to its parental SGC7901 cell line[30]. Also, some research has been directed at targeting the sites of such miRNAs. Motoyama *et al*[31] found that expression of the high mobility group A2 (HMGA2) non-histone chromosomal protein in cancerous tissues was significantly higher than in non-cancerous tissues and HMGA2 expression status was an independent prognostic factor. An inverse correlation between HMGA2 and let-7a was found in gastric cancer cell lines. These results suggested that HMGA2 is negatively regulated by the let-7 miRNA family in human gastric cancer. The miR-106b-25 cluster, up-regulated in a subset of human gastric tumors, is activated by E2F1 in parallel with its host gene, Mcm7. In turn, miR-106b and miR-93 regulate E2F1 expression, establishing a miRNA-directed negative feedback loop. The miR-106b-25 cluster is involved in E2F1 posttranscriptional regulation and may play a key role in the development of TGF beta resistance in gastric cancer[32]. Xia *et al*[30] assumed that miR-15b and miR-16 could play a role in the development of MDR in gastric cancer cells, at least in part by modulation of apoptosis via targeting BCL2.

In our experiment, miRNA-21 expression in sporadic gastric tumor tissues was remarkably up-regulated relative to the non-tumor tissues, which is consistent with existing reports. miR-21 has frequently been reported to be aberrantly overexpressed in diverse tumors, including gastric cancer[33] and other solid tumors[34]. In the past 3 years, at least 3 direct targets of miR-21 have been identified, with all of them being tumor suppressors: the PTEN phosphatase, the actin-binding protein tropomyosin 1 and the reversion-inducing-cystein-rich protein with Kazal motifs. Thus, this single miRNA provides a significant survival advantage to cells upon deregulation[35]. Despite these findings, the role and relevant pathway of miR-21 in gastric carcinogenesis is largely unknown.

In this study we also found a significant overexpression of miR-155 in sporadic gastric tumor tissue obtained from this Chinese population. Similar results were also found in other studies. Overexpression of miR-155, measured either at the primary or mature transcript level, was described in diffuse large B-cell lymphomas (DLBCL)[36]. A set of seven miRNAs, including miR-155, that were most differentially overexpressed in thyroid tumors *vs* hyperplastic nodules in surgical samples were validated and shown to provide high accuracy in the detection of thyroid cancer[37].

The miR-155 target genes have not yet been fully elucidated, but many discoveries have given hints to reveal the complete picture. Yin *et al*[38] found that miRNA-155 was an Epstein-Barr virus-induced gene that modulates Epstein-Barr virus-regulated gene expression pathways. Tumor protein 53-induced nuclear protein 1 expression is repressed by miR-155, and its restoration inhibits pancreatic tumor development[39]. The study of DLBCL found that miR-155 expression was associated with NF-kappaB activity. miR-155 expression segregates with specific molecular subgroups of DLBCL and it is highest in activated B-cell (ABC)-type lymphomas[36]. miR-155 also has an important role in the mammalian immune system, specifically in regulating T helper cell differentiation and the germinal center reaction to produce an optimal T cell-dependent antibody response. miR-155 exerts this control, at least in part, by regulating cytokine production. Highly specific knockouts of miR155 in mice resulted in multiple defects in adaptive immunity[40].

miR-145 has been regarded as a tumor suppressor gene[17],[41]. But, our data showed that there was no significant difference in miR-145 expression between cancerous gastric tissue and non-cancerous gastric tissue from the same patient. Down-regulation of miR-145 expression has been reported in breast cancer[42], primary liver cancer[43], colorectal cancer[44]-[46], chronic lymphocytic leukemia, B-cell lymphomas, and Burkitt lymphoma[47]. The treatment of human colon cancer cells with miR-145 caused growth arrest and miR-145 was identified as a miRNA that inhibits the growth of human cancer cells[48].

In the present study we found that expressions of miR-21, -145 and -155 were not correlated with such factors as age, degree of differentiation, histologic type, lymph node metastasis, or Hp infection. Many studies have been carried out to investigate the relationships between miRNAs and clinical diagnosis and prognosis of different tumors. Differences in the profiles of miRNAs between normal and pathological conditions might be promising as biomarkers in early diagnosis and prognosis[49]. A miR-200 miRNA cluster was reported as a prognostic marker in advanced ovarian cancer[50]. miRNA expression profiles of varying pancreatic tissues have identified a number of differentially expressed miRNAs that seemed to be able to differentiate between three tissues of clinical importance: normal pancreas, chronic pancreatitis, and pancreatic ductal adenocarcinoma[51]. Elevated expression of certain miRNAs, including miRNA-155, in pancreatic tumors is associated with poorer survival[52]. The miR-106a level was significantly associated with gastric tumor stage, size and differentiation, lymphatic and distant metastasis and invasion, and may be a potential biomarker in the diagnosis of gastric carcinomas[53]. All these data indicated that miR-155 was involved in the pathogenesis of gastric cancer. But, in the present study, we did not find such relationships between the expression of the miRNA studied and clinopathologies. This indicates that perhaps much more work is needed to be done to clarify these relationships because even in different individuals of the same race, the gene expressions may vary widely[25]. In this study, there was no significant difference in the miR-145 expression between gastric cancer tissue and paired non-cancerous tissue. This may indicate that miRNA-145 is expressed differently in different racial groups. Reports on the differential expression of genes among different racial groups have been presented for years[22]-[24].

It is difficult to find a miRNA with a manner of expression that accurately matches all aspects of a certain kind of cancer. After an analysis of 95 miRNAs, miR-92 was found significantly elevated in plasma of patients with colorectal cancer[54]. Our results also demonstrated that expression of miR-21 and miR-145, whether in gastric cancer tissue or in noncancerous tissue, had a higher abundance than that of miR-155.

Formalin-fixed paraffin-embedded (FFPE) tissue samples were used to carry out this experiment. Tetzlaff *et al*[55] also proved that FFPE tissue samples were reliable sources for studying miRNA expression. Hoefig *et al*[56] studied the expressions of 157 miRNAs in fresh frozen samples and parallel FFPE samples with real-time quantitative PCR, and the results showed that miRNA expression patterns were rarely affected by the FFPE treatment. Such methods allow us to access large data banks of archival material[57]. With the technique used here, we also obtained a series of reliable data, because the standard error of each group of Ct values was no more than 1 % of the respective mean Ct value. Therefore, results from the FFPE samples reflect the real miRNA expression information in the samples. Both this study and the referenced experiments prove that it is possible to use the most frequently preserved FFPE specimens on file in hospitals for further large-scale studies of miRNA expression in various diseases.
